# Prevalence and Risk Factors of Pediculosis in Primary School Children in South West of Iran

**Published:** 2018-12

**Authors:** Jalil NEJATI, Amir KEYHANI, Amir TAVAKOLI KARESHK, Hossein MAHMOUDVAND, Abedin SAGHAFIPOUR, Maryam KHORAMINASAB, Razieh TAVAKOLI OLIAEE, Seyed Mohammad MOUSAVI

**Affiliations:** 1. Health Promotion Research Center, Zahedan University of Medical Sciences, Zahedan, Iran; 2. Dept. of Medical Parasitology and Mycology, Kerman University of Medical Sciences, Kerman, Iran; 3. Infectious Diseases Research Center, Birjand University of Medical Sciences, Birjand, Iran; 4. Dept. of Medical Parasitology and Mycology, Lorestan University of Medical Sciences, Khorramabad, Iran; 5. Dept. of Public Health, School of Health, Qom University of Medical Sciences, Qom, Iran; 6. Dep. of Education Office, Andimeshk County, Ahvaz, Iran

**Keywords:** *Pediculosis capitis*, Risk factors, Children

## Abstract

**Background:**

Pediculosis or louse infestation is a public health problem in many developing countries where the WHO’s primary health-care program is inefficient and haphazard. The present study aimed to determine the prevalence of *Pediculus capitis* infestation and its related risk factors in the primary school children in Andimeshk, Dezful and Shoosh counties; Khuzestan Province, Iran.

**Methods:**

Overall, 28410 students in the age range of 7–11 years old in primary schools of North of Khuzestan Province, southern Iran were examined individually and privately under the flash light for all life cycle stages of lice or their nits in 2016. A questionnaire was filled for each school child before hair examination; then examination was carried out to detect head lice as well as eggs/nits.

**Results:**

Overall, 2995 students (10.5%) were infested with pediculosis. There was a significant difference in the prevalence of pediculosis among the boys and girls students. The prevalence of infestation was also significantly (*P*<0.05) higher in students of living in nomad tribes (23.8%) in comparison of rural (12.4%) and urban areas (6.5%).

**Conclusion:**

Several risk factors significantly (*P*<0.05) related to pediculosis included gender of female, nomad habitat, parents education, father’s occupation, having health staff, history of infestation and number of combing per day. Increasing awareness and training of teachers and relatives, as well as for improving standards of personal health, can significantly reduce the prevalence of pediculosis.

## Introduction

Public health is very important issue in any society so that the public health progress of society depends on its people health. One of the threatening society health is insect infestation that dispite of health promotion and advancement of medical services, it is still considered as a major health problem (1, 2).

Nowadays, large and busy cities with the marginalized and poor areas where have health care facilities often have numerous health problems. One of which is head lice infestation especially among primary school students and their families (3). The lice are tiny wing less and obligate, blood-sucking ectoparasite of humans that dorsoventrally flattened. It belongs to phylum Arthropoda, class insect and order Anoplura Systematically. There are three families of human lice: *Pediculus capitis* (head louse), *P. corporis* (body louse), and *Phtirus pubis* (pubic louse). They have been well-known as human ectoparasitites presented inhead, body and genital organs serface (4).

Head louse infestation is a public health problem in many developing countries where the WHO’s primary health-care program is inefficient and haphazard (5). Approximately 6–12 million people are infected by this insect in different areas of the world annually (6). The prevalence of head lice infestation is variable between less than 5% to over 40% among school children, respectively (7). Based on epidemiological studies in schools of world various countries have shown different frequency of pediculosis; 13.6% in Mexico (8), 26.6% in Jordan (9), 15.3% in South Africa (10), 23.32% in Thailand (11), 26.4% in Nigeria (12), and 28.3% in England (13).

Many studies in different areas of Iran in this regard has been done sporadically. For instance, frequency of head lice in Khomain city; central Iran 11.9% was estimated (14). Moreover, this health problem in primary schools of Qom province 7.6% was reported (15). “Totally, prevalence of head lice infestation in various areas of Iran indicate that total prevalence of infestation varied from 0.47% in Isfahan (center of Iran) to 27% in Sistan-Baloochistan Province (south-east of Iran)” (5).

Generally, the presence of one of the common three species of human lice (body, head or pubic lice) on any part of the human body is called pediculosis. According to this point that human head lice is eating blood several times a day and saliva injected into the body repeatedly, its toxic effects in infested people may cause fatigue, irritability, pessimism and feeling lazy mode and cause severe itching (16). This health problem can lead to depression, psychiatric disorders, academic failure and cause insomniain school-aged children (17). The common ways of transmission of head lice to humans is happen through contact with infected people via person to person directly andthrough close indirect contact lice carrying such as clothes, personal belongings, beds or furniture infested by nits (lice eggs) or adult lice.

The most effective strategies to human lice control is the use of shampoos containing pediculosides such as lindane and permethrin for treating infested people and also holding public education in communities to promote public health (18,19).

In recent years, no comprehensive study has been done on head lice infestation in Andimeshk, Dezful and Shush, north of Khuzestan Province, so the present study aimed to determine the prevalence of *P. capitis* infestation and related risk factors in the primary school children in Khuzestan Province, Iran.

## Materials and Methods

### Sampling

This descriptive cross-sectional study was performed in the primary school’s children of urban, rural and nomadic tribes in Andimeshk, Dezful and Shoosh counties; north of Khuzestan Province, Iran during Feb to Jun 2016. The study was carried out on 280 primary schools selected randomly, according to the most clusters. Overall, 28410 students (14150, 11721, 2539) people resident in urban, rural and nomadic tribes respectively) in the age range of 7–12 yr old were examined individually and privately under the flashlight for all life cycle stages of head lice such as nites/eggs, nymph or adult lice. The nomadic tribes from Shahre-Kord, Esfahan, Ilam and Shiraz move to this area at Sep to Apr of each year. These people have atleast three features, including tribal social structure, reliance on subsistence farming and nomadic lifestyles.

### Ethics approval

The present investigation was approved by Ethics Committee of Kerman University of Medical Sciences (Permit No. 93/324). However, written consent was obtained from all participants or their legal guardians after they had been fully informed of the nature of the study according to the code of ethics in the declaration of Helsinki protocol. The epidemiological data were collected via researcher made questionnaire and visual inspection on head hair was applied for detecting lice infestation and evaluating prevalence of this problem. The infested people had contacted with the head louse in one of its life cycle stages or presence of the egg/nit in the distance of 1.4 inches (less than 4 cm) from the scalp (20).

### Questionnaire and risk factors

We have considered some demographic data including age, gender, educational grade and residence. Moreover, information about hair condition and probable effecting factors such as occupational and educational levels, family size, living area, Having health staff, shered instruments, history of infestation, number of combing per dayand bathing per week using filling the standard check list conducted and approved by the Health Ministry of the Islamic Republic of Iran.

### Statistical analyses

Analytical and descriptive statistics was carried out using SPSS 17.0 software (SPSS Inc., Chicago, IL, USA). Descriptive statistics were reportedin terms of percent (for categorical) and mean (SD) (for continuous) variables. Chi-square test was applied to access the univariate association between independent variables and outcome. *P*<0.05 was considered to be statistically significant.

## Results

Overall, 28410 students aged 7–11 were included in the present study. Most participants were girls (53.7%), living in urban areas (49.8%). Out of 28410 primary school students, 2995 students (10.5%) were infested with *P. capitis* in one of its life cycle stages (egg/nit, nymph or adult) or presence of the egg/nit in the distance of less than 4 cm from the scalp. The students with 7 years old had the highest infestation rate (16.45%). There was no significant difference between the prevalence of pediculosis and students age groups (Table 1). From 2995 infested children, 26.6% and 73.4% were boys and girls, respectively. There was a significant difference between the prevalence of pediculosis and gender of students (*P*<0.05) (Table 1).

**Table 1:** Socio-demographic features of the study subjects and the prevalence of pediculosis capitis among primary schools of Kuzestan Province, Iran 2016

***Features***	***Level***	***Total***	***Pediculosis capitis frequency***		***P-value***
			***N***	***%***	
Age (yr)	7	4697	773	16.45	0.574
	8	6143	798	12.99	
	9	7011	526	7.50	
	10	5460	499	9.14	
	11	5099	399	7.82	
Gender	Male	13141	797	6.06	<0.002
	Female	15269	2198	14.39	
Educational grade	I	4777	766	16.03	0.522
	II	6043	831	13.75	
	III	7120	513	7.20	
	IV	5510	520	9.43	
	V	4960	365	7.36	
Family size	3 persons	4102	331	8.07	0.822
	4 persons	9110	966	10.60	
	5 persons	7043	723	10.26	
	6 or more than 6 persons	8155	975	11.95	
Living area	Urban	14150	931	6.58	<0.041
	Rural	11721	1459	12.44	
	Nomads	2539	605	23.82	
Having health staff	Yes	19720	1224	6.20	<0.001
	No	8690	1771	20.38	
Shared instrument	Yes	9119	1359	14.90	0.584
	No	19291	1636	8.48	
Father’s occupation	Unemployed or died	5341	898	16.81	<0.049
	Self-employed	16295	1644	10.09	
	Governmental-employed	6774	453	6.68	
Father’s education	Illiterate or died	2110	633	30.00	<0.001
	Initial education	13015	1204	9.25	
	University education	13285	1158	8.71	
Mother’s education	Illiterate or died	3917	889	22.69	<0.002
	Initial education	15877	1021	6.43	
	University education	8616	1085	12.59	
History of infestation	Yes	1814	711	39.19	<0.001
	No	26596	2284	8.58	
Number of combing per day	None	2530	455	17.98	<0.041
	Once	12150	1009	8.30	
	Twice	13730	1131	8.23	
	Three and more	4309	400	9.28	
Bathing per week	Once or less	16340	1983	12.13	0. 764
	Twice	10841	910	8.39	
	More than twice	1229	102	8.29	

The highest infestation in the study found in first grade (16.03%). However there was no observed relation between head louse infestation and educational grade. In term of living area, 931(6.58), 1459(12.44) and 605(23.82) cases in urban, rural areas and nomadic tribes were recorded, respectively. The prevalence of infestation was also significantly (*P*<0.041) higher in students who living in nomad tribes (23.8%) in comparison with rural (12.4%) and urban areas (6.5%). The majority of human head lice infested students 1771 (20.38%) had no access to health staff for receiving primary health services. Moreover, 14.90% out of students who had shared instrument were infested with head lice. In term of family size, 975 (11.95%) lived in families with six or more siblings. Although cases had increased in crowded families but the difference between the groups was not statistically significant. The prevalence of head lice infestation was higher in people who had parents with low educational level, their father was unemployed or died and having history of infestation previously (*P*<0.01) (Table 1). 17.98% of head lice infested people were not combing their hair daily and 12.13% of them bathed once or less per week. The result of analysis showed a significant relationship between head louse infestation and the number of combing per day (*P*<0.05) (Table 1).

## Discussion

*P. capitis* is an infestation that affects mainly children. Depending on the socio-economic setting, these infestations may have an impact on a large proportion of a population (21). The current investigation was designed to evaluate the prevalence of head lice infestation and its related risk factors in primary school children in Khuzestan Province, Iran. Prevalence of pediculosis among students in some Middle Eastern and other regional countries ranged from 4.2%–78% (22–26). In Iran, the prevalence of pediculosis was 581 per 100,000 population that varied from 1/100,000 in Shiraz city to 8,303/100,000 in Kerman City. The highest prevalence of pediculosis was documented in the southeastern cities of Iran, such as Kerman, Bandar Abbas, Zahedan and Zabol (27). Thetotal infestation rate in the present study was 10.5% that is one of the lowest rates among the reported results from the Middle Eastern and other regional countries. Several studies have reported the prevalence of pediculosis in various parts of Iran; for example, a study preformed on 5–9 yr old children in Tabriz showed that it was 5.7% (28). Other study on primary schools in Babul, exhibited that the prevalence of infection was 2.2%, while it was 6.85% in Hamadan province (29, 30).

The finding of a national study on 21 Iranian provinces (31) showed that out of 11.1% head lice infestation prevalence rate 5.7% belonged to Sistan and Balochestan Province where the infestation is the most prevalent among the whole population of the country (20). Similar to all previous studies in Iran and other parts of the world, we found the prevalence of pediculosis in school boys was lower than school girls. This significant difference could beattributed to behavior patterns between boys and girls which affected transmission rates like girls’ clothing. Furthermore, girls generally have longer hair as compared to boys and longer hair require better grooming and combing. Moreover, the suitability of female’s hair as a breeding place for head lice, covering of the female’s hair by scarf and so on.

In the present study, students in urban areas had lower prevalence of pediculosis than rural areas and nomad tribes, probably attributed to better hygiene, because more often urban school have health teacher or supervisor.

The obtained findings of this study in line with other studies conducted in Kerman (32), Hamadan (33), Turkey (29, 34) and Saudi Arabia (35) showed the prevalence of pediculosis among children with educated parents were lower than that among children with uneducated parents; indicating that literacy was an important factor in the prevalence of infection. Here, we found an association between the pediculosis and the parent’s occupation among the primary school children. These findings similar to the other studies indicated that low socioeconomic status significantly increased the rate of head pediculosis (32, 36).

Low educational level of their parents was a risk factor in head lice infestation and most of students who had parents with elementary level of education were infested. Several studies have been conducted mainly in relation of socio- demographical status of people such as their educational level on head lice infestation (37, 38).

## Conclusion

The obtained findings of the present study showed more prevalent among children with gender of female, nomad habitat, illiterate parents, unemployed fathers. Our suggestion is that socio-economic levels and hygienic conditions should be improved for successful treatment of pediculosis capitis, through increasing awareness of parents by the educational programs.

## Ethical considerations

Ethical issues (Including plagiarism, informed consent, misconduct, data fabrication and/or falsification, double publication and/or submission, redundancy, etc.) have been completely observed by the authors.
